# BRCA 1/2 mutations and risk of uterine cancer: a systematic review and meta-analysis

**DOI:** 10.1186/s12863-024-01189-y

**Published:** 2024-01-31

**Authors:** Faezeh Zakerinasab, Qumars Behfar, Reza Parsaee, Reza Hossein Zadeh, Elaheh Foroughi, Amirhesam Amirbeik, Ghazalehsadat Ahmadi

**Affiliations:** 1 Mashhad University of Medical Sciences, Mashhad, Iran; 2 Department of Neurology, Faculty of Medicine and University Hospital Cologne, University of Cologne, Cologne, Germany; 3 Master Student in Molecular Genetics, Transplant Research Center, Shiraz University of Medical Sciences, Shiraz, Iran; 4 Student’s Research Committee, Faculty of Medicine, Mashhad University of Medical Sciences, Mashhad, Iran; 5 School of Medicine, Isfahan University of Medical Sciences, Isfahan, Iran; 6 Student Research Committee, School of Medicine, Mashhad University of Medical Sciences, Mashhad, Iran

**Keywords:** BRCA1, BRCA2, Mutation, Uterine cancer, Endometrial

## Abstract

**Purpose:**

In this study, we aim to investigate the association between BRCA1/2 mutation and uterine cancer incidence.

**Material and method:**

We systematically searched three databases including PubMed, Scopus, and Google Scholar up to August 2023; and reviewed 23 cohorts and cross-sectional studies to explore the association between BRCA1/2 mutations and uterine cancer incidence.

**Results:**

This systematic review comprised a total of 21 cohort studies and 2 cross-sectional studies after the screening process. According to meta-analysis the prevalence of the BRCA1/2 gene in patients with uterine cancer was 0.02 (95%CI = [0.01,0.03], *P* < 0.01, I^2^ = 94.82%)

**Conclusions:**

Our meta-analysis investigates a 2% prevalence of BRCA1/2 mutation in patients with uterine cancer. Patients with BRCA1/2 mutations might be more conscious of uterine malignancies.

## Background

Uterine cancer risk in BRCA1/2m women and its significance in the BRCA mutated condition are still beyond controversy. The similarities between serous ovarian and uterine cancers, especially serous carcinomas, have prompted researchers to look for shared pathogenetic traits as well as hereditary factors. Even though the particular gene sequence implicated is mostly unclear, 10% of the cases of endometrial cancer are found to have positive family histories, suggesting a genetic tendency [1]. BRCA1 is located in chromosome 17q21, whereas BRCA2 is located in chromosome 13q12, which are both autosomal dominant tumor suppressor genes involved in DNA damage repair prior to cell replication [2, 3]. BRCA1 and BRCA2 mutation carriers’ risk of developing uterine cancer is still unknown due to inconsistent results from various studies that may have been impacted by past tamoxifen treatment. However, the majority of research has indicated an approximately two-fold increase in risk compared to the general population [4]. A higher risk of endometrial cancer, and especially uterine serous carcinoma in BRCA1m women, was verified by some writers [5–7], but not by others [8, 9]. 11,847 BRCA1 variant carriers participated in global cohort research of the Breast Cancer Linkage Consortium that discovered a significant two- to three-fold increase in the risk of endometrial cancer. The fact that BRCA1 variant carriers may use tamoxifen, which is known to raise the risk of endometrial cancer [10, 11].

Endometrial cancer develops in the inner layers of the uterus from a glandular epithelium layer that covers the luminal surface and secretes substances that are essential to normal periods and developmental stages of development. Endometrial cancer is a common cancer influencing the female reproductive organs in higher-income states [12]. In patients with no metastatic disease, five years of overall survival ranges from 74 to 91% [13]. An aggressive form of endometrial cancer (EC), uterine serous carcinoma (USC) accounts for 5–10% of all uterine carcinomas and represents over 40% of EC-related death [14]. Numerous studies have demonstrated an elevated risk of developing EC, particularly in gBRCA1 carriers, with the largest risks being seen in an aggressive subtype of EC called serous-like ECs [8, 14]. Others, however, failed to notice this elevated risk or blamed it on prior tamoxifen therapy rather than the gBRCA1 mutation [9, 15]. And also, some other studies revealed an elevated risk of developing EC, particularly in those who carried the mutation in the BRCA1 gene, with the serous-like form of EC showing the highest reported risk [16]. These BRCA1-associated endometrial cancers are associated with an unfavorable outcome [16] and it requires additional studies to confirm these findings. The aim of this systematic review and meta-analysis is to determine how BRCA1/2 affects uterine cancer and provide answers to these concerns.

## Method

This systematic review and meta-analysis investigate the effect of BRCA 1/2 mutations on the risk of uterine cancer. The research protocol was registered on the Open Science Framework (OSF) platform.

### Search strategy

We searched PubMed, Scopus, and Google Scholar databases to find any studies which demonstrated the effect of BRCA 1/2 mutations on the risk of uterine cancer up to August 1, 2023. For any additional eligible studies, reference lists of identified systematic reviews and included studies were manually checked. The search strategy of this systematic review and meta-analysis is available in Table 1. As the first step, the title and abstract of the screened articles were reviewed by one of the researchers after removing duplications.

**Table 1:** Search strategy for current systematic review and meta-analysis

Search engine	Search strategy	Additional filter	Result
PubMed/Medline	((“Uterine Neoplasms“[Mesh]) OR (“Endometrial Neoplasms“[Mesh]) OR (“Genital Neoplasms, Female“[Mesh]) OR (“Uterine Cervical Neoplasms“[Mesh]) OR (“Carcinoma, Endometrioid“[Mesh]) OR (“Atypical Squamous Cells of the Cervix“[Mesh]) OR (“Sarcoma, Endometrial Stromal“[Mesh]) OR (“Endometrial Stromal Tumors“[Mesh])) AND ((“BRCA2 Protein“[Mesh]) OR (“BRCA1 Protein“[Mesh]) OR (“BRCA1 Protein / genetics”) OR (“BRCA2 Protein / genetics”))	English, 2020	668
Scopus	((TITLE-ABS-KEY (uterine AND neoplasms) OR TITLE-ABS-KEY (endometrial AND neoplasms) OR TITLE-ABS-KEY (uterine AND cervical AND neoplasms) OR TITLE-ABS-KEY (endometrial AND stromal AND tumors) OR TITLE-ABS-KEY (endometrial AND carcinoma)) AND PUBYEAR > 2013 AND PUBYEAR < 2024) AND ((TITLE-ABS-KEY (brca2 AND protein) OR TITLE-ABS-KEY (brca1 AND protein)) AND PUBYEAR > 2013 AND PUBYEAR < 2024)	English, 2014	598
Google scholar	All intitle: BRCA Uterine All intitle: BRCA Endometrial Also, we checked the recent Systematic reviews references manually.	English	105

### Study selection

Cross-sectional studies, prospective cohorts, and randomized controlled trials (RCTs) that evaluated the effect of BRCA1/2 mutations on the risk of uterine cancer (uterine carcinoma and endometrial cancer) were included. Articles that didn’t match inclusion criteria or data that were not announced or case reports, editorials, and reviews were excluded because they didn’t provide sufficient data of methodological quality. In conclusion, a single rate meta-analysis and diagnostic meta-analysis have been reported.

### Data extraction and quality assessment

Two reviewers (EF, AA), extracted the necessary information based on the pre-defined criteria. The quality of the studies was checked by two reviewers (EF, AA) separately.

### Statistical analysis

Data related to the relationship between BRCA 1/2 mutations and the risk of uterine cancer were extracted from included studies.

A randomized meta-analysis model, as a result of the heterogeneity of study results, has been implemented in order to incorporate effect sizes. For the estimation of variance between study pairs, a method known as DerSimonian and Laird [17] was applied which is applicable to both within and intra-study differences. Heterogeneity between included studies was evaluated using Cochran’s Q test and I^2^ statistic, and I^2^ more than 50% was considered as heterogeneity. Subgroup analyses were planned to explore potential sources of heterogeneity, including study design (cohort vs. cross-sectional), menopausal age categories (early vs. late), and geographic location. In order to assess the differences between subgroups, interaction tests have been performed. A sensitivity test was performed to assess the robustness of the results. In order to assess their impact on the overall effect size estimate, the exclusion of studies with a high risk of bias of more than 50% has been carried out. To evaluate the study’s publication bias, a visual evaluation of the symmetry of the flow chart and Bagg and Egger regression tests have been carried out. SPSS version 22 was used to conduct all the statistical analyses and *p*-values less than 0.05 were considered statistically significant.

## Results

### Literature search

**Fig. 1:**
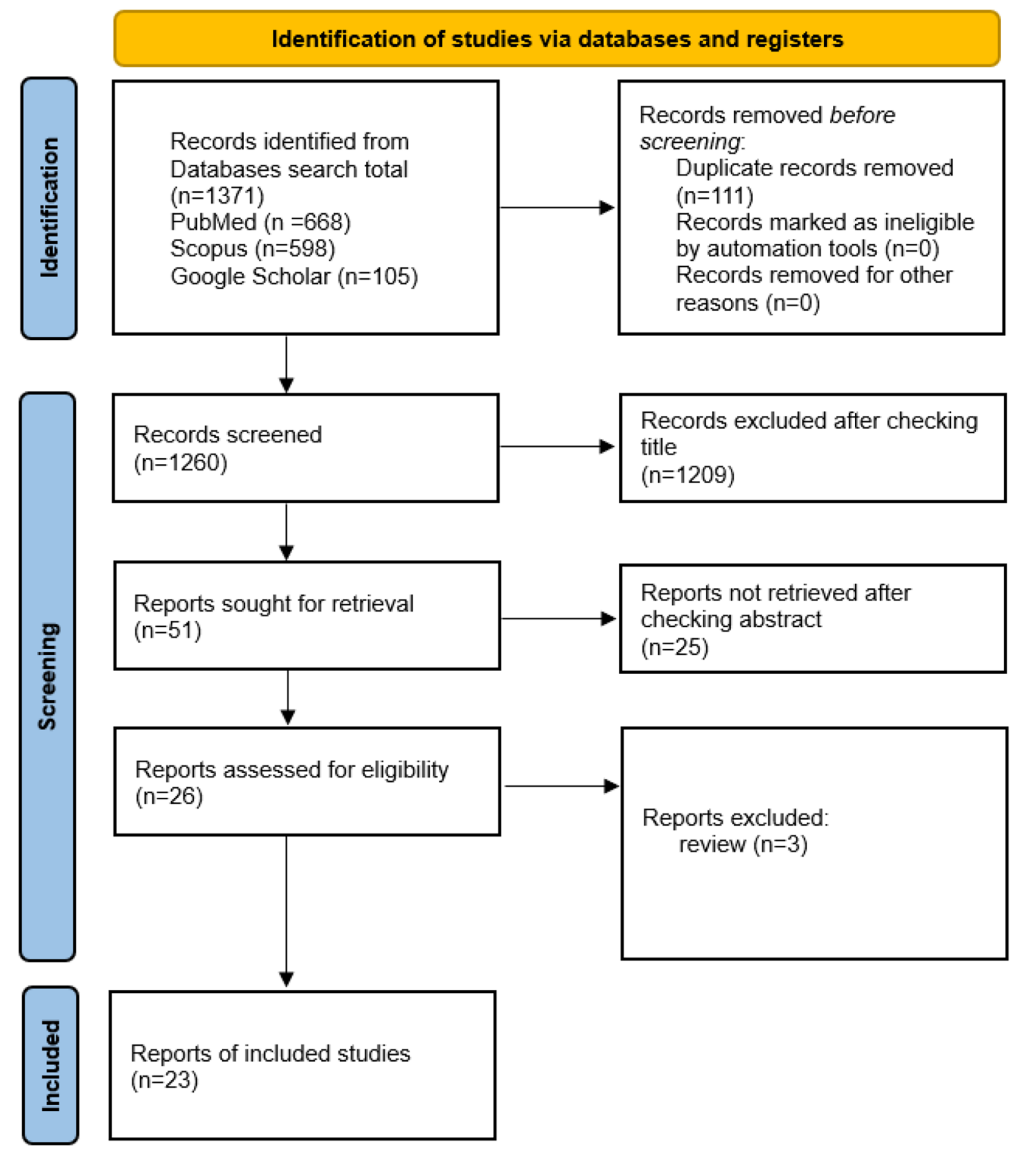
PRISMA diagram for this present systematic review and meta-analysis

A total of 1371 records were found overall by searching databases. 1260 papers were assessed after 111 duplicates were eliminated, and 1209 were excluded based on the titles. 25 findings were excluded after the papers’ abstracts were reviewed. A full text for 26 records was collected and 3 publications were excluded according to the study type review. Finally, 23 full-text articles were included in the systematic review after the screening process. Figure 1(PRISMA flow chart of study identification) reports specifics about the literature search findings. Studies that evaluated the relevance of BRCA1/2 mutations in individuals with uterine and endometrial cancer were all included [1, 5, 7–10, 15, 18–33].

### Patient characteristics and methodological aspects of the included studies

This systematic review included almost 26,000 patients in total, who were all evaluated. The included studies on the incidence of BRCA mutation in endometrial cancer patients had a patient population ranging from 7 to 3623, and the research on the incidence of endometrial cancer in BRCA mutation carriers had a patient population ranging from 828 to 5980. 3.3 to 14 years were covered by the median follow-up. This systematic review comprised a total of 21 cohort studies and 2 cross-sectional studies. Six researches included Jewish women in Israel [1, 7, 20, 27, 28, 31]. Seventeen studies were remained, of which 8 studies were conducted in United States [5, 18, 19, 21, 25, 26, 32, 33], 2 in Australia [22, 24], 2 in United Kingdom [10, 23], 2 in Canada [15, 29], 2 in Netherlands [9, 30], and 1 in France [8]. Table 2 demonstrated the key characteristics of eligible studies on the prevalence of BRCA mutation in patients with endometrial cancer and on the incidence of endometrial cancer in BRCA mutation patients. The prevalence of BRCA mutation varied from 0% [18] to 27.2% [27] in patients with uterine cancer, and the currency of uterine cancer ranged from 0.53% [23] to 1.87% [30] in BRCA1/2 mutation carriers according to the baseline table.

**Table 2:** The key characteristics of included studies of BCRA mutations and uterine cancer

Author [ref]	Year	country	Study design	Follow up duration	Participants	Age	mutations	Coincidence of uterine cancer & mutation (relative frequency)	
								BRCA1	BRCA2
Beiner (29)	2007	Canada	cohort	3.3 years	857 women carry a *BRCA1* or *BRCA2* mutation	45–70 (54.4)	six women were diagnosed with endometrial cancer	4/857	2/857
Bruchim (28)	2010	Israel	cohort	76 months	31 Jewish patients with USC	56–79 (66)	• 4 were BRCA2 (6174delT) carriers • 2 each carried the BRCA1 mutations (185delAG and 5382insC)	2/31	4/31
Burkett (26)	2019	United States	cohort	NR	109 patients (62% were endometrioid)	(47.5)	12.8% were sBRCA+		
Frey (21)	2017	United States	cohort	NR	Four hundred and fifty-four patients/ (96%) of tested patients were female / (26, 6%) reported a personal history of uterine cancer/	25–91 (52)	Among the 138 Ashkenazi Jewish patients Only two of the 20 mutations were in BRCA1/2 (10%)		
Frimer (18)	2016	United States	cohort	NR	7 consecutive patients with paired tumor and non-tumor USC samples in our institutional tumor repository	65–85 (75)	There were no BRCA1 or BRCA2 mutations reported	17/49	13/49
Hecht (19)	2014	United States	Cross-sectional	-	27 cases (All women diagnosed between 2007 and 2012 with USC in a hysterectomy specimen were included)	48–92	• Only for 5 patients the BRCA1 status was known. • Loss of BRCA1 expression: 4 (14.8%) 23 women (85%) had no personal history of breast cancer and one of them showed loss of BRCA1	2/3	0/3
Johnatty (24)	2021	Australia	cohort	NR	EC patients (n = 5292)/ 3623 patients were tested for BRCA1/2 variations	17–88	• Isolated EC (N = 1619 N Variant BRCA1: 12 (0.7) N Variant BRCA2: 12 (0.7) • EC & FH EC (N = 507) N Variant BRCA1: 2 (0.4) N Variant BRCA2: 3 (0.6) • EC & concur/subseq BC (N = 686) N Variant BRCA1: 8 (1.2) N Variant BRCA2: 15 (2.2) • EC & concur/subseq BC & FH EC (N = 163) N Variant BRCA1: 2 (1.2) N Variant BRCA2: 1 (0.6) • EC & prior BC (N = 548) N Variant BRCA1: 11 (2.0) N Variant BRCA2: 11 (2.0) • EC & prior BC & FH EC (N = 100) N Variant BRCA1: 5 (5.0) N Variant BRCA2: 2 (2.0)	40/5292	44/5292
Jonge (30)	2021	Netherlands	cohort	ended at the date of EC diagnosis	5980 *BRCA1/2* (3788 *BRCA1*, 2151 *gBRCA2*, 41 both *BRCA1/BRCA2*) and 8451 non-*BRCA1/2* mutation carriers		• EC (58 = 20.53%) BRCA1/2 (BRCA1: 44(12.53%) / BRCA2: 14(8.23%)) and 33 non-BRCA1/*2* mutation carriers • Endometrioid (35 = 16.85%) BRCA1: 27(10.27%) BRCA2: 8(6.77%) • Serous-like (19 = 1.95%) BRCA1:15(1.19%) BRCA2: 4(0.78%)	44/5980	14/5980
Kadan (20)	2018	Israel	cohort	14 years	64 patients (14 BRCA mutation carriers and 50 noncarriers)	47–79	• BRCA1 mutation (185delAG or 5382insC): 9 BRCA2 mutation (6174delT); 5		
Kitson (23)	2020	UK	cohort	NR	2609 women (1350 BRCA1 and 1259 BRCA2)		14 cases of endometrial cancer in women (1350 BRCA1 and 1259 BRCA2) mutation carriers		
Lee (22)	2017	Australia	cohort	9.0 years	828 mutation carriers) BRCA1 mutation: 438 BRCA2 mutation; 390)	34–52 (43)	• 5 incident cases of UC BRCA1: 3 BRCA2: 2	3/828	2/828
Biron-Shental (27)	2006	Israel	cohort	NR	22 Jewish patients with USPC	56–79 (71.8)	six BRCA1e2 germline mutation carriers (27%) as follows: three with BRCA2-6174delT, two with BRCA1- 185delAG, and one with BRCA1-5382insC mutation. T	3/22	3/22
Sun (25)	2016	United States	Cross-sectional	5.2 years	Fresh endometrial tissue was obtained from 97 cases (type I EC: 49/ endometrial atypical hyperplasia: 20)	•	• Atypical endometrial hyperplasia group: 3/20 (15.00%) BRCA1 • EC group: Endometrioid adenocarcinoma: 9/45 (20.93%) Non-endometrioid adenocarcinoma: 1/4 (33.33%)	18/49	0/49
Barak (1)	2010	Israel	Cohort	-	289 Jewish women with EC/ 251/289 patients (86.8%) had type I carcinoma with 245 (84.7%)—endometrioid-type/	27–89	• BRCA1*185delAG (n = 4) BRCA2*6174delT (n = 1) mutations none of 34 women with type II EC carried any BRCA1/BRCA2 mutations	4/289	1/289
Shu (5)	2016	United States	Cohort	5.2 years	1083 mutation carriers (BRCA1:727 and BRCA2: 453)	40.2–59.5 (45.6)	uterine cancer cases: 8	4/1083	1/1083
Segev (15)	2013	Canada	Cohort	5.7 years	4456 mutation carriers (BRCA1:3536 and BRCA2: 920)		uterine cancer cases: 17	13/4456	4/4456
Reitsma, Welmoed (9)	2013	Netherlands	Cohort	6 years	315 mutation carriers (BRCA1:201 and BRCA2: 144)	32–78 (50)	uterine cancer cases: 2		
Thompson, Deborah (10)	2002	UK	Cohort	NR	2245 mutation carriers (BRCA1:2245 and BRCA2: 0)		uterine cancer cases: 11		
Levine (31)	2001	Israel	Cohort	12 years	99 consecutive Ashkenazi Jewish patients with endometrial carcinoma		three *BRCA* founder mutations (185delAG and 5382insC in *BRCA1* and 6174delT in *BRCA2*		
Long (32)	2019	US	Cohort	NR	1170 patients		• BRCA1: EC type 1: 1/849 (0.12%) EC type 2: 3/321 (0.93%) USC: 1/135 (0.74%) • BRCA2: EC type 1: 3/849 (0.35%) EC type 2: 0/321 (0%) USC: 0/135 (0%)	5/1170	3/1170
Laitman (7)	2019	Israel	Cohort	32,774 women-years of follow up	2627 eligible mutation carriers (1463 *BRCA1*, 1154 *BRCA2* mutation carriers, 10 double mutation carriers)	45–77	uterine cancer cases: 14		
Lavie (33)	2000	US	Cohort	-	12 women with uterine serous papillary carcinoma	56–77	one 185delAG mutation and one 5382insC mutation.		
Saule (8)	2018	France	Cohort	4.8 years	369 BRCA 1 or 2 mutation carriers who underwent RRSO		• Endometrial carcinoma: 2 Serous endometrial carcinoma: 2	2/369	0/369

### Main findings

**Fig. 2:**
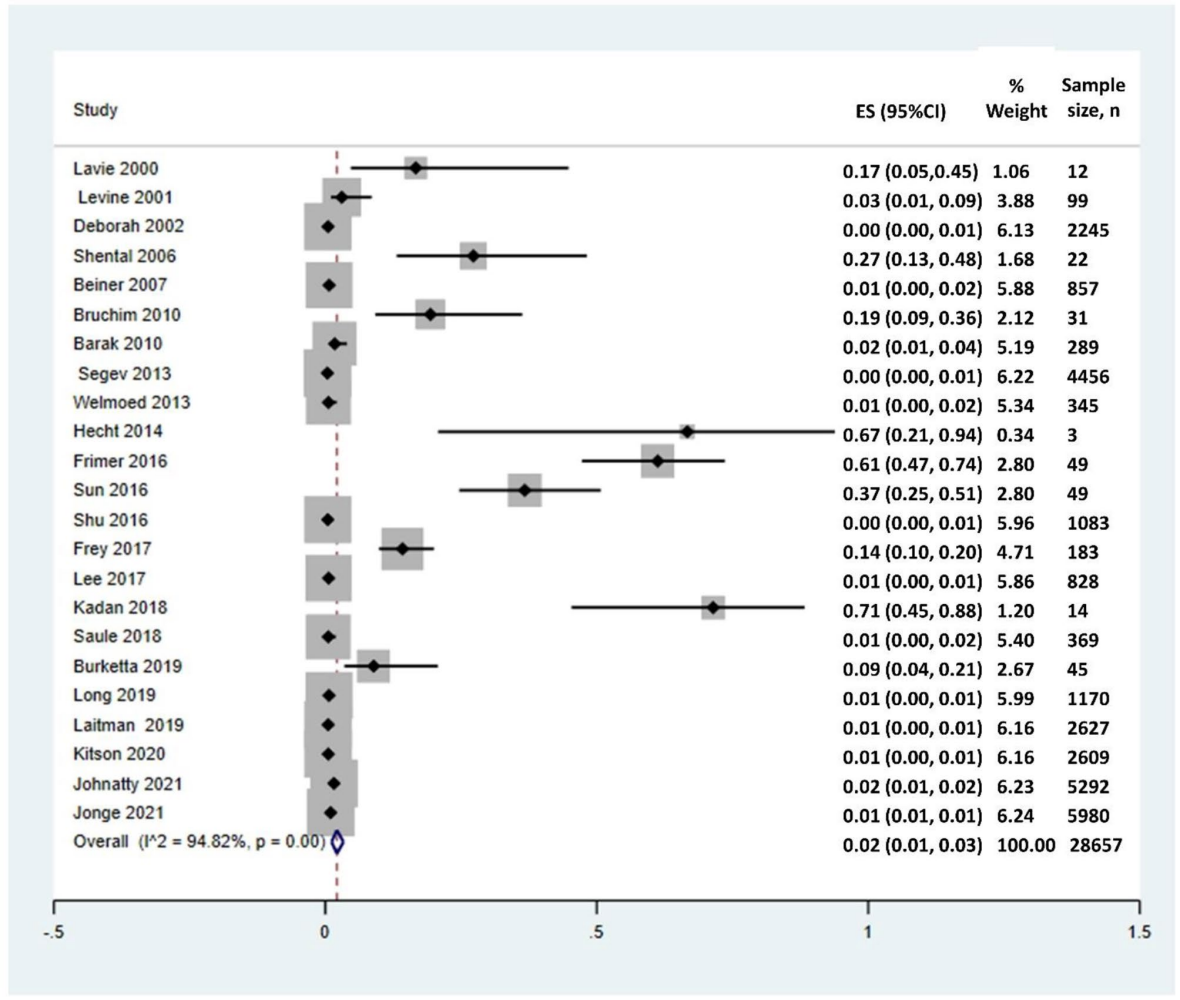
Forest plot diagram for included studies

**Fig. 3:**
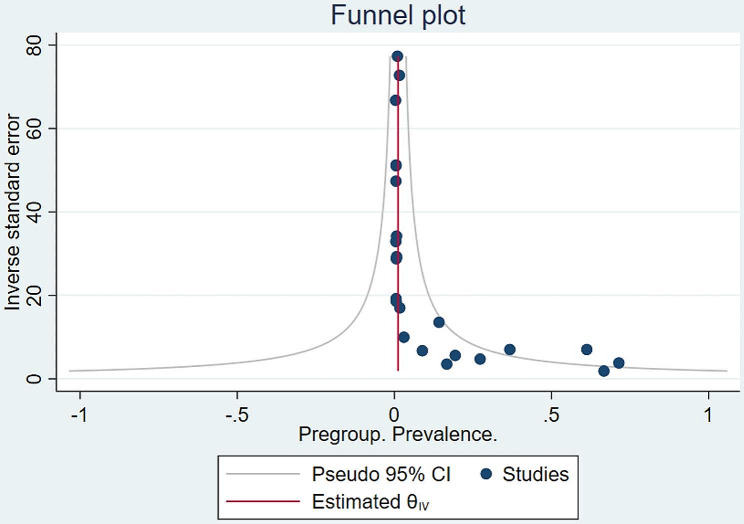
Funnel plot diagram for included studies

According to meta-analysis (Fig. 2) the prevalence of the BRCA1/2 gene in patients with uterine cancer was 0.02 (95%CI = [0.01,0.03], I^2^ = 94.82%, *p* < 0.01). In proportion to Funnel plot (Fig. 3) and sensitivity analysis were also provided no studies were excluded according to their results.

## Discussion

The clinical significance of BRCA1 and BRCA2 mutations has been covered in numerous research. To draw a conclusion from these data, there is still much work to be done [34]. Gene mutations are thought to be the main cause of about 5% of cases of endometrial cancer [35]. Characterizing somatic genetic changes in uterine cancer has received a lot of attention in the last ten years, but uterine cancer’s molecular causes are not yet well understood. Such uterine cancers exhibit similar molecular and morphological characteristics, indicating the potential of a connection to hereditary breast and ovarian cancer and the BRCA1 mutation [19]. Furthermore, we did a reverse analysis of prospective research to determine the risk of uterine cancer in BRCA mutation carriers. We summarized all of the research that examined uterine cancer patients for BRCA mutation as well as the prevalence of uterine cancer in BRCA mutation women in this systematic review. Finally, our findings showed that the prevalence of BRCA mutations in patients with uterine carcinoma is 2%. Numerous studies have found that UPSC patients have a high prevalence of BRCA mutations and that BRCA mutation carriers have a higher risk of developing UPSC [5, 7], but some other results do not corroborate these results [8, 9, 19, 23]. Compared to the general population, women with a deleterious BRCA1 or BRCA2 mutation had a roughly 2.5-fold greater risk of getting uterine cancer, according to the cohort study on the rate of BRCA mutation in uterine cancer. Despite the fact that this was statistically insignificant, the SIR was 2.87 for BRCA1 mutation carriers and 2.01 for BRCA2 mutation carriers, to be more specific [22]. These findings prompted us to gather information and perform subgroup analysis on 21 previously published cohorts and 2 cross-sectional studies in order to determine if BRCA mutations might contribute to the pathogenesis of uterine cancer and also to improve these patients’ outcomes. One of the studies just reported the prevalence of BRCA1 in uterine cancer cases [19] and one of them didn’t segregate the BRCA1 and BRCA2 prevalence [26]. Therefore, the population usually overlaps. For example, eight studies chose patient from the United States [5, 18, 19, 21, 25, 26, 32, 33], six from Israel [1, 7, 20, 27, 28, 31], and two from Australia [22, 24].

A significant retrospective study from the Breast Cancer Linkage Consortium revealed an elevated chance of uterine cancer for BRCA1 mutation carriers, but weren’t for BRCA2 mutation carriers (RR = 2.65; 95% CI: [1.69,4.16]; *P* < 0.001) [10, 36]. In this cohort study of 1,083 women, there were five incident cases of serous/serous-like endometrial carcinoma that occurred after RRSO, four in BRCA1 mutation carriers, and one in BRCA2 mutation carriers. However, when tumor subtypes were analyzed, there was statistically significant increased risk of serous carcinomas in BRCA1 mutation carriers (observed to expected ratio of 22.2, 95%CI: [6.1,56.9], *P* < 0.001). There was no evidence of a causal relationship between BRCA1/2 pathogenic variants and serous endometrial cancer; carriers of these variants did not have a higher risk of developing the disease, and there were no pathogenic types found in the BRCA1/2 genes in the tumor cells from 15 random cases of serous endometrial cancer. These encouraging results are in line with those of Lee et al., who did not observe an increase in serous or endometrioid endometrial cancer in their small Australasian population, and Levine et al., who reported a relative risk of endometrial cancer of 0.75 (95% CI = [0.24, 2.34], *p* = 0.6) in 199 Ashkenazi Jews with BRCA1/2(BRCA1 SIR = 2.87, 95%CI = [0.59,8.43], *p* = 0.18 / BRCA2 SIR = 2.01, 95%CI = [0.24,7.30], *p* = 0.52) [22]. In the study using case-case approach, pathogenic mutations in BRCA1 and BRCA2 were linked to higher chances for uterine/EC (odds ratio [OR]: 3.1, 95%CI: [1.6,5.7]). Their findings show that BRCA1 and PALB2 pathogenic mutations are more common in EC patients with prior BC and a family history of EC when using a case-case approach [24]. Another study suggested that one out of the three prevalent BRCA germline mutations that are recognized in the Jewish population were carried by 27% of our patients with a diagnosis of USPC. The known mutation rate (2.5%) for the general Jewish community is considerably less than this mutation incidence. Only 22 Jewish women was the population of the cohort research and conclusions may be conflicting. Considering that the Jewish population has a distinctive carrier pattern for the three typical BRCA1e2 germline mutations, this population bias may be a reasonable explanation for the discrepancy between the contradicting results; although the Lavie et al.‘s [37] findings and this study’s total quantity of BRCA germline mutations were similar, but the location of the mutations is different [27].

In order to describe the discrepancy in results, we should consider several reasons: First, all of the cohort studies were limited to the population of a region and that may cause race and genetic panel differences. Second, in some studies BRCA1/2 germline mutation was limited by the small number of women with genetic testing results or were included small population [18, 19]. Third, Tamoxifen taking and personal history of breast cancer are effective cause that didn’t screened in all studies. And forth, follow up durations varied. Proof of advantages is unquestionably required to balance these additional risks given the increased potential morbidity linked to more complex surgery. It is currently unclear whether or not there is a distinct benefit in particular subgroups of BRCA pathogenic variant carriers because neither this study nor which have been published before contained data on body mass index (BMI), making it unable to account for this in analyses [23].

### Limitations

When analyzing the results of the current study, a few restrictions must be taken into attention. First, linguistic bias can be an issue because only English-language articles were chosen. Second, because the eligible studies under consideration all were conducted in various locations, recall bias and selection bias were unavoidable. Third, it may be biased because favorable results were more likely to be published than negative discoveries. Fourth, Patients with a significant family history may be examined for potential mutations, although some patients may pay less attention to their family history and avoid the tests [3].

The direction of bias depends on the magnitude and direction of each bias alone. For example, if the selection bias is high in the publication of studies with favorable results and people with a positive family history, the prevalence of BRCA 1/2 in uterine cancer is overestimated. Contrariwise, if the recall bias prevails in remembering the positive family history, the prevalence of this mutation is underestimated. Both of these directions highlight the need to interpret the data with caution. However, in this study we used sensitivity tests to assess the bias and no significant bias was found, which reduces the possible bias rate, leading to validity og the results.

## Conclusion

Our meta-analysis investigates 2% prevalence of BRCA1/2 mutation in patients with uterine cancer. Patients with BRCA1/2 mutations might be more conscious of uterine malignancies. Our findings might help physicians enhance therapy options for USC patients by including targeted therapies and preventing and genetic guidance. Large scaled observational studied are needed to further support this articles results.

## Data Availability

Not applicable.

## Abbreviations

EC: Endometrial cancer
USC: Uterine serous carcinoma
OSF: Open Science Framework
RCTs: Randomized controlled trials
BMI: Body mass index
